# Identification and analysis of differently expressed transcription factors in aristolochic acid nephropathy

**DOI:** 10.1265/ehpm.23-00245

**Published:** 2024-05-21

**Authors:** Yi-Feng Wu, Zhi-Yao Tang, Yi-Xuan Deng, Kun Liu, Xu-Rui Gu, Guang-Liang Zhou, Yu-Jie Huang, Xiao-Qing Lin, Lin-Yun Zhou, Xiao-Cong Zuo

**Affiliations:** Department of Pharmacy, The Third Xiangya Hospital, Central South University, Changsha 410000, China

**Keywords:** Aristolochic acid nephropathy, ATF3, c-JUN, Transcription factor

## Abstract

**Background:**

Aristolochic acid nephropathy (AAN) is a rapidly progressive interstitial nephropathy caused by Aristolochic acid (AA). AAN is associated with the development of nephropathy and urothelial carcinoma. It is estimated that more than 100 million people worldwide are at risk of developing AAN. However, the underlying mechanisms driving renal deterioration in AAN remain poorly understood, and the treatment options are limited.

**Methods:**

We obtained GSE27168 and GSE136276 series matrix data from the Gene Expression Omnibus (GEO) related to AAN. Using the R Studio environment, we applied the limma package and WGCNA package to identify co-differently expressed genes (co-DEGs). By GO/KEGG/GSVA analysis, we revealed common biological pathways. Subsequently, co-DEGs were subjected to the String database to construct a protein-protein interaction (PPI) network. The MCC algorithms implemented in the Cytohubba plugin were employed to identify hub genes. The hub genes were cross-referenced with the transcription factor (TF) database to identify hub TFs. Immune infiltration analysis was performed to identify key immune cell groups by utilizing CIBERSORT. The expressions of AAN-associated hub TFs were verified in vivo and in vitro. Finally, siRNA intervention was performed on the two TFs to verify their regulatory effect in AAN.

**Results:**

Our analysis identified 88 co-DEGs through the “limma” and “WGCNA” R packages. A PPI network comprising 53 nodes and 34 edges was constructed with a confidence level >0.4. ATF3 and c-JUN were identified as hub TFs potentially linked to AAN. Additionally, expressions of ATF3 and c-JUN positively correlated with monocytes, basophils, and vessels, and negatively correlated with eosinophils and endothelial cells. We observed a significant increase in protein and mRNA levels of these two hub TFs. Furthermore, it was found that siRNA intervention targeting ATF3, but not c-JUN, alleviated cell damage induced by AA. The knockdown of ATF3 protects against oxidative stress and inflammation in the AAN cell model.

**Conclusion:**

This study provides novel insights into the role of ATF3 in AAN. The comprehensive analysis sheds light on the molecular mechanisms and identifies potential biomarkers and drug targets for AAN treatment.

## Graphical Abstract

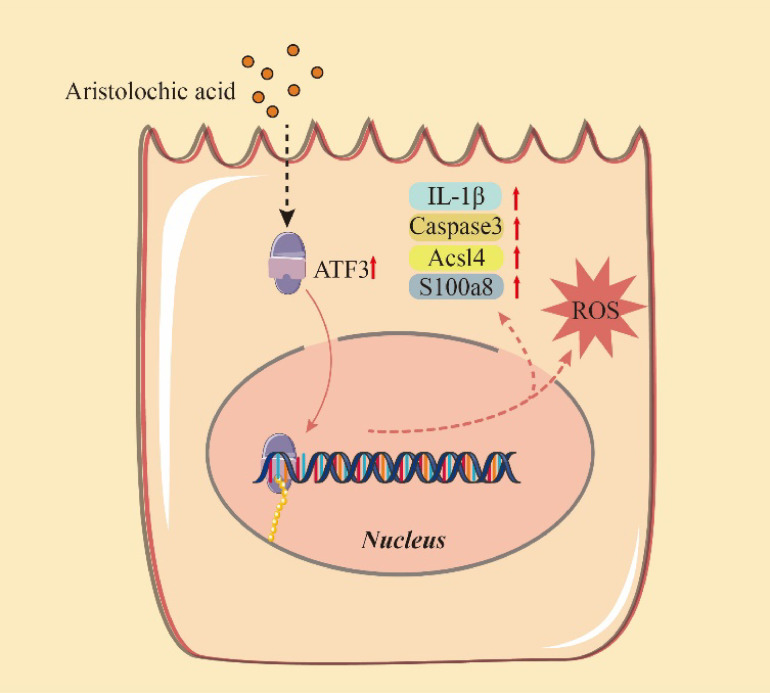

AA can cause the abnormal increase of ATF3 expression in renal tubular epithelial cells, which promotes the enhancement of intracellular reactive oxygen species, inflammation-related factors and oxidative stress.

## 1. Background

Aristolochic acid nephropathy (AAN) arises from exposure to aristolochic acid (AA), nitrophenanthrene carboxylic acids found in Aristolochiaceae family plants [1]. AA, mainly comprised of 8-methoxy-6-nitro-phenanthro-(3,4-d)-1,3-dioxolo-5-carboxylic acid (aristolochic acid I, AAI) and 6-nitro-phenanthro-(3,4-d)-1,3-dioxolo-5-carboxylic acid (aristolochic acid II, AAII), contributes to AAN [1]. AAI, the key AA component through reductive activation and forming covalent DNA adducts, induces kidney damage triggering cell cycle arrest, and apoptosis [2]. Despite International Agency for Research on Cancer (IARC) classifying AA as highly carcinogenic and toxic in 2002, it was still available online [3]. Consequently, AAN cases are prevalent globally, with over 100 million people at risk of AAN, leading to rapid progression to end-stage renal disease (ESRD) [4, 5]. Also, AAN is associated with more than 40% of the prevalence of urothelial carcinoma [4]. However, there are no strict criteria for diagnosing AAN, and also no specific therapy for treating AAN. Therapies for acute AAN are steroids and angiotensin-receptor blockers (ARBs) / angiotensin-converting enzyme inhibitors (ACEIs) [6]. Therapies for chronic kidney disease (CKD) are limited to renal replacement therapy and transplantation [7, 8]. To address this challenge, it is crucial to explore the pathogenesis of AAN and effectively prevent and treat the disease.

The exact mechanisms of AAN are not fully characterized. Studies have demonstrated that apoptosis, oxidative stress, and inflammation are involved in the occurrence and development of disease [9]. Transcription factors (TFs) are proteins that play a significant role in coordinating gene expression [10]. Previous researches had indicated that TFs are crucial for the occurrence and development of acute kidney injury (AKI). Studies have implicated specific TFs, such as IRF4 and ATAT1, had a role in the development of AAN [11, 12]. However, the critical TFs and specific mechanisms underlying AAN are not yet fully understood [1]. Therefore, this study aims to identify the hub TFs involved in AAN, which may facilitate a better understanding of the disease’s mechanisms and help us to identify more effective therapeutic targets.

At present, sequencing technology, together with integrated bioinformatics analysis, has been widely used to identify disease-related genes and drug targets and to analyze complex disease pathogenic mechanisms [13–16]. In this study, we downloaded corresponding RNA data from cell and mice AAN models from GEO databases and identified differently expressed genes (DEGs) by comparing the AAN models with normal controls. The DEGs were then subjected to gene ontology (GO) functional annotation and Kyoto Encyclopedia of Genes and Genomes (KEGG) pathway analysis. Through protein-protein interaction (PPI) network analysis and the cytoHubba plugin, we identified the hub genes, which were then overlapped with TF databases to find the hub TFs. Furthermore, we validated the hub TFs using real-time polymerase chain reaction (PCR) analysis and Western blotting, and further confirm their effect by using siRNA. The identified hub TF may serve as potential diagnostic biomarkers and therapeutic targets for preventing the occurrence and development of AAN.

## 2. Method

### 2.1 GEO datasets selection and DEGs screening

Two GEO datasets, namely GSE27168 and GSE136276 were obtained in the GEO database (https://www.ncbi.nlm.nih.gov/geo/). GSE27168 includes microarray data from human renal tubular epithelial cell line (HK-2) cells after the addition of Aristolochic acid 24 and 48 hours, with three samples in each group. GSE136276 consists of microarray data from the kidneys of C57BL/6 mice treated with 3.5 mg/kg body weight of AAI and sacrificed each day, with six samples in each group. The details of the data are shown in Table 1. Then, we performed difference analysis using the limma package between the control and AAN groups in GSE27168 and GSE136276 datasets. Genes displaying an absolute log fold change (log FC) above 1 and a P-value below 0.05 were categorized as DEGs. Using the GSE136276 gene expression data, we constructed a scale-free network via the R package WGCNA. The top 50% of genes with the highest median absolute deviation (MAD) were then selected. We determined an optimal “soft” parameter (b) for adjacency calculation based on co-expression similarity. Module eigengene dissimilarity was computed, and a suitable dendrogram cut line merged multiple modules. The module most correlated with AAN was identified, and its genes were intersected with the DEGs from the limma package. Results were visualized using R packages anRichment and ggplot2.

**Table 1:** Information for selected microarray datasets

**GEO Accession Total samples**	**Selected samples Platform**	**Source tissue**	**Sample type:**
GSE136276 10 Samples	GPL23038	Kidney	5 WT_AAI
			5 WT_Water
GSE27168 12 Samples	GPL570	HK-2 cells	3 24 h Control
			3 24 h AAI
			3 48 h Control
			3 48 h AAI

### 2.2 Enrichment analysis

Initially, we mapped DEGs from mice to their human homologs. Commonly co-upregulated and co-downregulated DEGs across all three groups were identified using R. Subsequently, utilizing the “clusterprofiler” R package, we conducted Gene Ontology (GO) enrichment analysis encompassing biological process (BP), cellular component (CC), and molecular function (MF) categories, elucidating functional implications of co-upregulated and co-downregulated DEGs. For pathway analysis, we employed the ClueGO plugin within Cytoscape. Selected pathways contained a minimum of 3 genes per node, with pathway network connections determined by a moderate Kappa score (P < 0.05 indicated significance). Enriched pathways for all DEGs were recognized using the KEGG and Reactome databases.

### 2.3 PPI network construction and hub TF screening

The PPI network is used to analyze the functional interactions between proteins. In this study, we uploaded the previously screened co-DEGs to the STRING database (https://string-db.org/) to construct potential PPI relationships, and a composite score >0.4 was considered statistically significant. The PPI networks were mapped using Cytoscope 3.7.2 (https://cytoscape.org/). We used maximal clique centrality (MCC) algorithms to evaluate the hub genes. Then we overlapped the TF database with hub genes we got previously to get hub TFs.

### 2.4 Immune analysis algorithm

We employed the R package mMCPcounter to analyze immune cell proportions, including 8 immune cell types and 2 stromal cell types. The resulting heatmap illustrated immune cell abundance. Spearman non-parametric correlations were employed to assess the relationship between key TFs and immune-infiltrating cells.

### 2.5 Cell culture

The HK-2 cells were purchased from Zhong Qiao Xin Zhou Biotechnology Co., Ltd., Shanghai, China. Cells were cultured at 37 °C in a 5% CO2 humidified environment using DMEM/F12 media (Gibco, Thermo Fisher Scientific) supplemented with 10% fetal bovine serum (FBS, Gibco). AA (Sigma-Aldrich; Merck KGaA, USA) was dissolved in PBS to 0.5 mmol/L, and cells were treated with a final concentration of 40 µmol/L AA. The Cell Counting Kit 8 (CCK8, Apexbio) assay was used to detect cell viability under the manufacturer’s instructions.

### 2.6 Animals

Animal experiments were approved by Central South University’s Animal Ethics Committee (approval no. CSU-2022-0206). Male C57BL/6 mice, 7 weeks old, were sourced from Central South University (Changsha, China). Mice were housed under controlled conditions (12:12 light/dark cycle, 25 °C, humidity) and provided freely access to a normal chow diet and distilled water. Following a week of acclimatization, mice were randomly assigned to two groups: AAN (n = 6) and control. Control mice received 0.9% normal saline, while the AAN group received intraperitoneal injections of AA at 5 mg/kg/day for 6 days. The renal tissues and blood samples were collected for subsequent analysis.

### 2.7 Western blotting assay

Renal tissues and HK-2 cells were lysed using RIPA lysis buffer. Protein lysates were separated by SDS–PAGE and transferred to PVDF membranes. Membranes were probed with anti-ATF3 (1:1000 dilution, ABclonal, A13469), anti-c-Jun (1:1000 dilution, ABclonal, A11378), anti-ACSL4 (1:1000 dilution, abclonal, A16848) and anti-IL1β (1:1000 dilution, ABclonal, A16288), anti-IL6 (1:1000 dilution, ABclonal, A0286), anti-S100A8 (1:1000 dilution, ABclonal, A12018), anti-Caspase-3 (1:1000 dilution, cell signaling technology, 9662S) and anti-tublin (1:10000 dilution, ZEN-BIOSCIENCE, R380648) antibodies at 4 °C overnight. Subsequently, HRP-labeled secondary antibody (1:10000 dilution, ZEN-BIOSCIENCE, 550094) was applied at room temperature for 1 hour. Western blot results were analyzed using Image Lab software.

### 2.8 Real-time PCR analysis

Total RNA was isolated from renal tissues and HK-2 cells using Trizol (TransGen Biotech, Beijing, China), following the manufacturer’s instructions. Subsequently, 1 µg of RNA was reverse transcribed into cDNA using the RT kit (TransGen Biotech, Beijing, China). Real-time PCR was carried out with gene-specific primers (listed in Table 2) using the SYBR green mixture kit. Relative mRNA expression of hub genes was normalized to β-actin, used as an internal control.

**Table 2:** The primers used for PCR

**Gene**	**GenBank entry**	**Primer**	**Sequence (5′ → 3′)**
β-actin	NC_000007.14	HF	CCACCATGTACCCAGGCATT
		HR	CGGACTCATCGTACTCCTGC
GPX4	NC_000019.10	HF	CCGCTGTGGAAGTGGATGAAGATC
		HR	CTTGTCGATGAGGAACTGTGGAGAG
ATF3	NC_000001.11	HF	GCCGAAACAAGAAGAAGGAGAAG
		HR	CAAATGCTGCTTCTCGTTCTTGA
JUN	NC_000001.11	HF	CCTGCCCAGTGTTGTTTGTAAAT
		HR	ACTCTTTCAACTCCACCTAGATAGC
IL-1β	NC_000068.8	HF	CTGGACCTCTGCCCTCTGG
		HR	TCCATGGCCACAACAACTGA
IL-6	NC_000007.14	HF	CACCGGGAACGAAAGAGAAG
		HR	CGCTTGTGGAGAAGGAGTTCAT
SMAD3	NC_000015.10	HF	CAGCCGGTTTGGATTACAGG
		HR	GAGTCAAAGTCCCTGCTCCT
β-actin	NC_000071.7	MF	GAGTCTGAAGTCGGGACCAC
		MR	TTTTCCTCTTGCCTCCTGAA
ATF3	NC_000067.7	MF	AGTCAGTTACCGTCAACAACAGA
		MR	CTCAGCATTCACACTCTCCAGTT
JUN	NC_000070.7	MF	TGCATGGACCTAACATTCGATCT
		MR	GCCCTCCCTGCTTTGTGTTAAG

F: forward; R: reverse

### 2.9 Gene silencing

The siRNA of ATF3, c-JUN, and their matched scramble control were purchased from TsingKeBiotechnologyCo., Ltd., (Shanghai, China). In brief, cells were transfected with 100 nmol/L of the indicated siRNA using a transfection reagent (TsingKeBiotechnologyCo., Ltd., TSV405) according to the manufacturer’s protocol. The HK-2 cells were pre-incubated with siRNA for 24 h and co-incubated with AA for 48 h. The sense of ATF3: GAGAAACCUCUUUAUCCAA, and antisense of ATF3: UUGGAUAAAGAGGUUUCUC. The sense sequence for c-JUN siRNA: CAAUAUUCAAGCGCGACAA, and antisense sequence: UUGUCGCGCUUGAAUAUUG.

### 2.10 Determination of reactive oxygen species (ROS) content in cells

Reactive Oxygen Species Assay Kit (DHE) (APPLYGEN, Beijing, China, C1300-2) was used for the assessment of the total ROS. HK-2 cells were incubated with 10 µM DCFH-DA in an incubator (37 °C, 5% CO_2_) for 30 min in the dark. After washing 3 times with PBS, the fluorescence of cells was analyzed by using an Operetta^®^ High Content Imaging System (PerkinElmer, Massachusetts, USA).

## 3. Results

### 3.1 Identification of DEGs in HK-2 cells and C57BL/6 mice

**Fig. 1:**
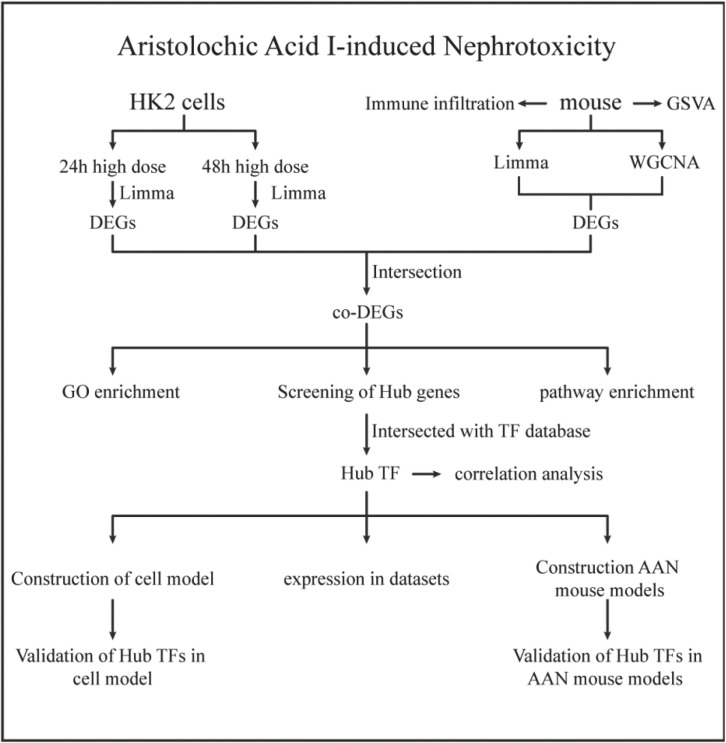
Workflow: Limma (linear models for microarray data) was used to identify DEGs (differentially expressed genes) from the GEO (Gene Expression Omnibus Series) dataset. TF, transcription factor; GSVA, gene set variation analysis; AAN, aristolochic acid nephropathy.

As illustrated in Fig. 1, we retrieved two mRNA expression profiles from the GEO database. These profiles included HK-2 cells treated with AA for 24 h and 48 h, as well as samples from a mice model. To conduct a comprehensive analysis, we employed limma and WGCNA methods to identify DEGs. Subsequently, we intersected the DEGs with a TF database to pinpoint hub TFs. These hub TFs were further validated using both HK-2 cells and animal model data.

**Fig. 2:**
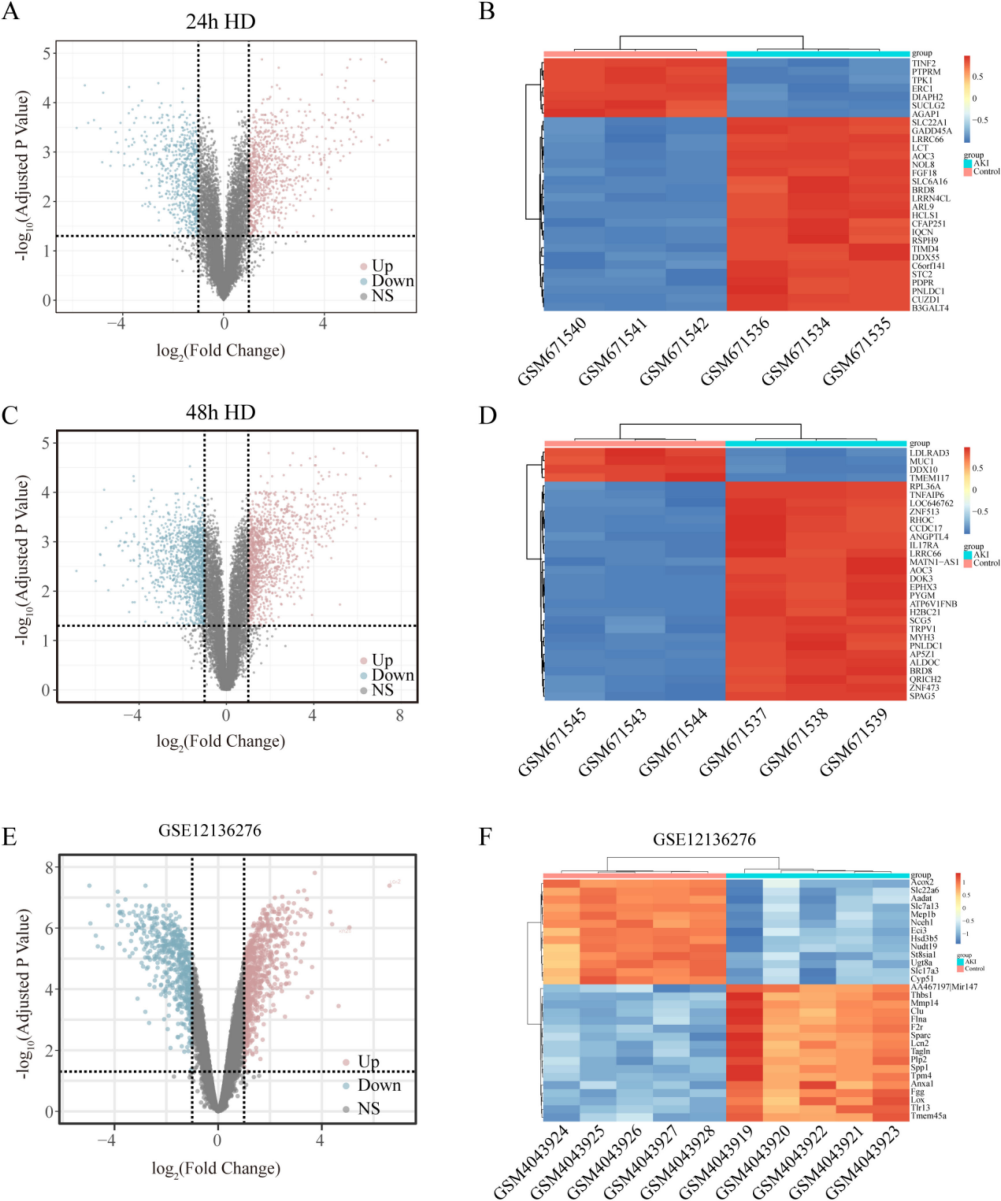
Identification of DEGs from the AAN model of HK-2 cells (GSE27168) and mice model (GSE136276). (A) Volcano plot of 24 h AAN cell model DEGs. (B) Heatmap of top 30 DEGs with highest P-Values in 24 h model. (C) Volcano plot of 48 h model DEGs. (D) Heatmap of top 30 DEGs with highest P-Values in 48 h model. (E) Volcano plot of mice model DEGs. (F) Heatmap of top 30 DEGs with highest P-Values in mice model. Color transitions from red to blue denote upregulation to downregulation.

In the treated with 24 h AA group comparison with control (GSE27168), we identified 1783 DEGs (Fig. 2A, B). In the treated with 48 h AA group comparison with control (GSE27168), we identified 2955 DEGs (Fig. 2C, D). The results of DEGs were visualized in the form of a Volcano plot. To explore the DEGs in vivo, we analyzed the AAN animal model datasets (GSE136276). A total of 2,705 DEGs were identified from the animal datasets. The top 30 genes with low P-values were presented as a heatmap. The results of DEGs were also visualized in the form of a Volcano plot. (Fig. 2E, F).

### 3.2 Identification of differential expression genes in animal model

**Fig. 3:**
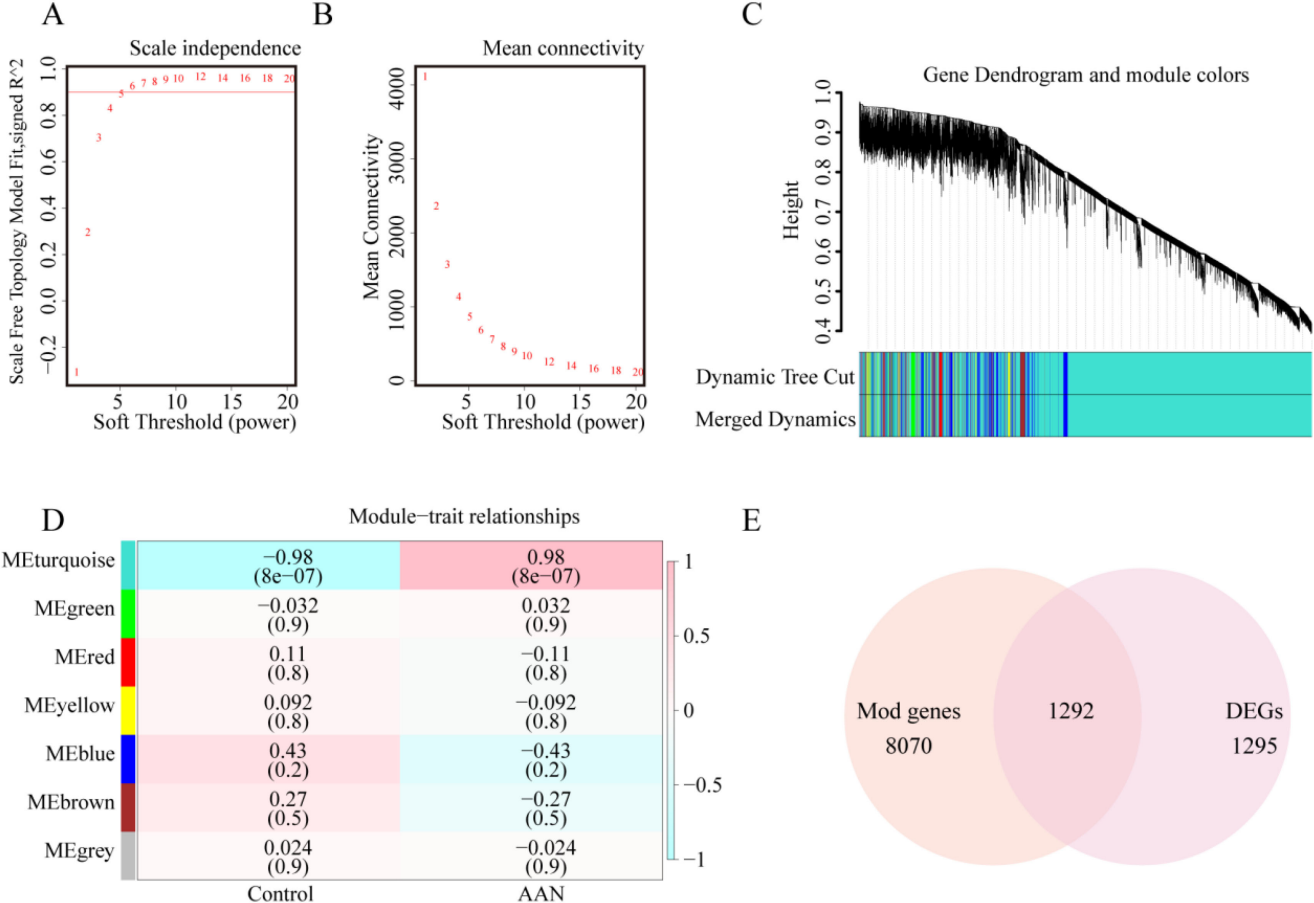
Identification of DEGs in the animal model. (A, B) Soft threshold established based on scale independence and mean connectivity. (C) Hierarchical clustering tree representing gene co-expression modules in distinct colors. (D) Heatmap illustrating module associations with AAN. The turquoise module demonstrated a significant correlation with AAN. Numbers above and below brackets indicate the correlation coefficient and P-value, respectively. (E) Venn diagram depicting the intersection of genes in turquoise module and DEGs.

To investigate AAN-related modules through WGCNA, we selected a “soft” threshold of β = 5 (ensuring scale-free R2 > 0.8) based on scale independence and average connectivity (Fig. 3A, B). After calculating the adjacency matrix and constructing a hierarchical clustering tree, modules were obtained. To simplify the network, modules with similarity above 0.75 were merged (Fig. 3C). In Fig. 3D, the turquoise module (8070 genes) exhibited the strongest correlation with AAN (correlation coefficient = 0.98, p = 8e-07), deemed pivotal for subsequent analysis. Moreover, the intersection of AAN associated genes from WGCNA and DEGs from the limma package yielded 1292 genes (Fig. 3E).

### 3.3 Gene enrichment and identification of hub TFs

**Fig. 4:**
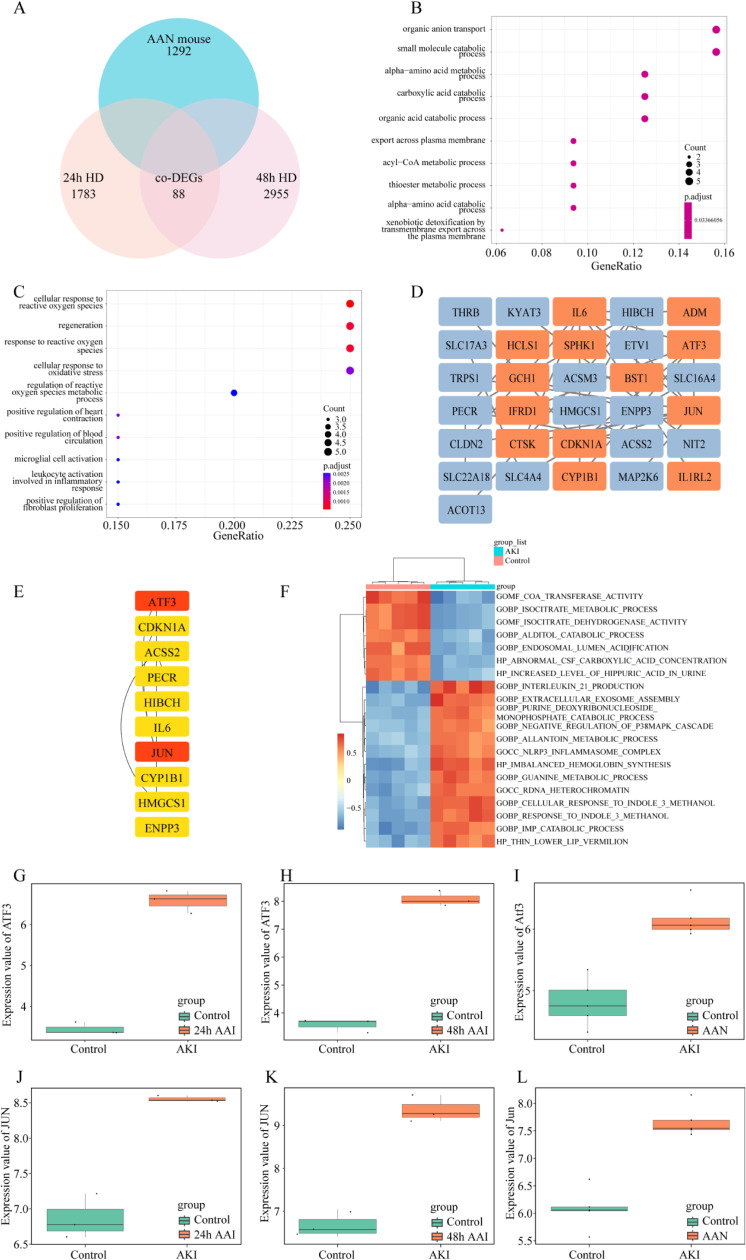
Identification of hub TFs (A) Venn diagram displaying the overlap of DEGs between cell and mice AAN models. (B) GO enrichment (BP, CC, MF) for up-regulated co-DEGs. (C) GO enrichment (BP, CC, MF) for down-regulated co-DEGs. (D) Construction of PPI network. (E) Hub genes are identified via the MCC algorithm. (F) GSVA-derived clustering heatmap of GSE136276’s differently expressed genes (DEGs). (G–I) ATF3 expression in cell and mice datasets. (J–L) c-JUN expression in cell and mice datasets. BP, biological process; CC, cellular component; MF, molecular function.

**Fig. 5:**
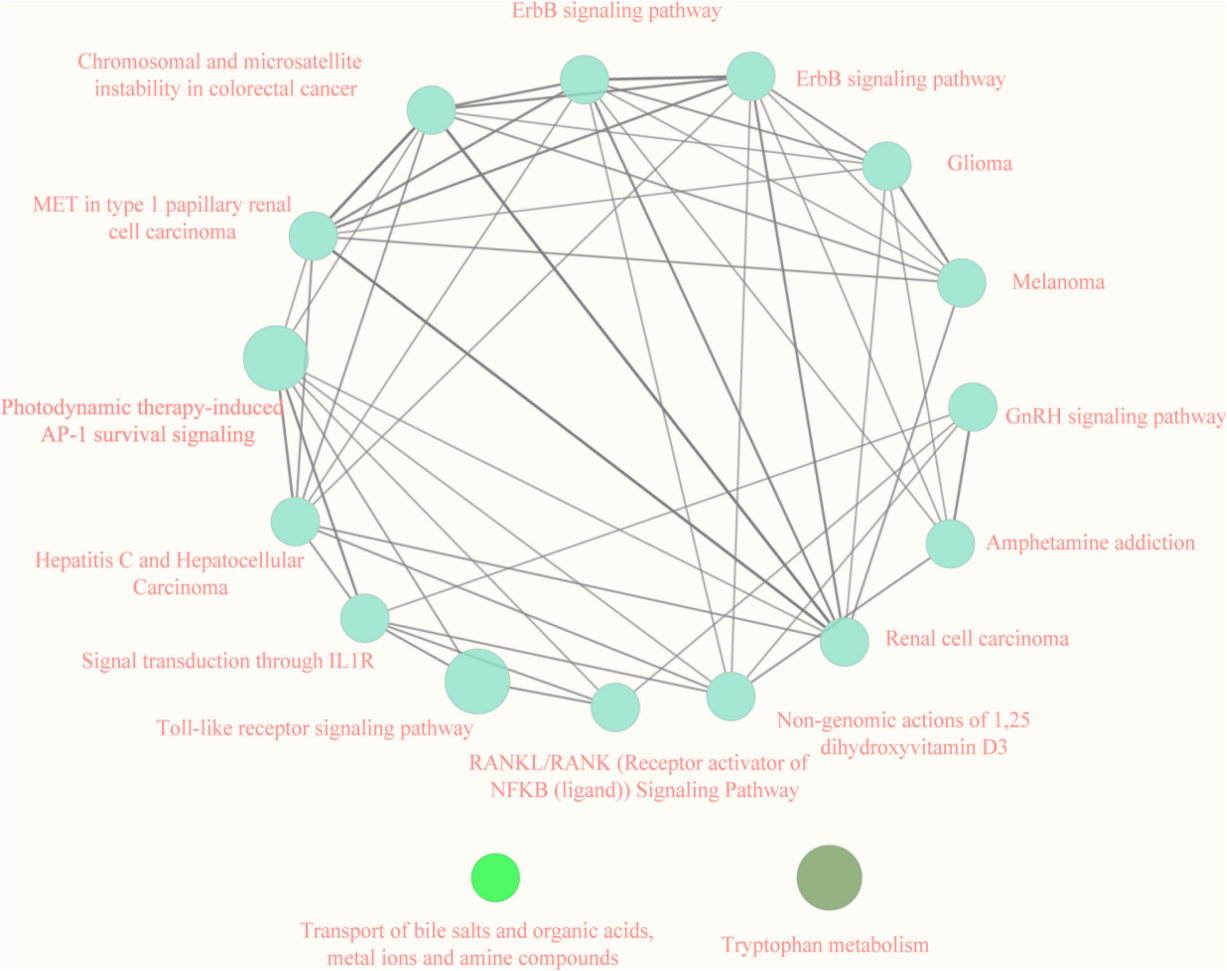
Functional pathways showed by KEGG/Reactome Pathway Database.

We overlapped the DEGs in both the HK-2 cells model and the mice model, and obtained 88 co-DEGs (Fig. 4A). To explore the relationship between these 88 genes and the pathogenesis of AAN, enrichment analysis was performed based on these genes. The co-upregulated genes were mostly enriched in organic anion transport, and small molecule catabolic process (Fig. 4B). However, the co-down-regulated genes were associated with cellular response to reactive oxygen species, regeneration, and response to reactive oxygen species (Fig. 4D). Gene Set Variation Analysis (GSVA) was conducted to investigate the biological functions. As depicted in Fig. 4F, several pathways exhibited changes. Specifically, pathways related to transferase activity, isocitrate metabolic process, and isocitrate dehydrogenase activity showed a decrease. On the other hand, signaling pathways associated with interleukin 21 production, extracellular exosome assembly, and purine deoxyribonucleoside displayed an increase. To identify the most significant clusters of the DEGs, the PPI network of co-DEGs was constituted by STRING, then the PPI network was visualized on these co-DEGs by Cytoscape. MCODE plugin was used to explore the most highly connected modules and visualized in Fig. 4D. The top 10 genes were identified as hub genes by the MCC algorithm. Then we obtained a gene set including over 1,600 TFs from https://doi.org/10.1016/j.cell.2018.01.029 and intersected them with the hub genes to screen out hub-TFs. Eventually, c-JUN and ATF3 were identified as hub TFs. We presented the consistent expression of these two hub TFs of different datasets by boxplots (Fig. 4E–J). The Kyoto Encyclopedia of Genes and Genomes (KEGG)/Reactome pathway database was used to figure out the functional roles of the robust DEGs. It showed that the robust DEGs mainly enriched in the “Toll-like receptor signaling pathway” and “Photodynamic therapy-induced AP-1 survival signaling pathway (Fig. 5).

### 3.4 Immune cell infiltration analysis

**Fig. 6:**
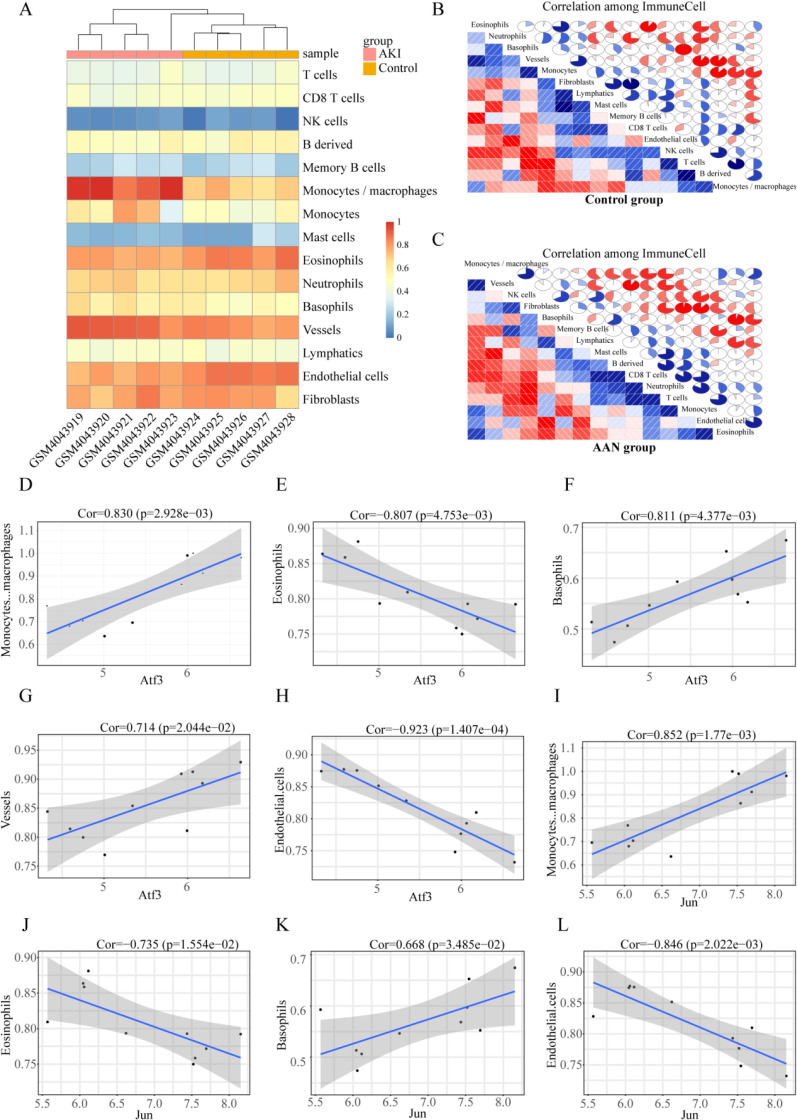
Immune cell infiltration analysis. (A) Heatmaps of the immune cell abundance in GSE136276; (B) Correlation matrixes of immune cells in the control group, red indicates negative correlations and higher values indicate higher correlations. Both horizontal and vertical axes demonstrate immune cell subtypes. (C) Correlation matrixes of immune cells in the control group; (D–H) The correlation analysis of ATF3 with Monocytes, basophils, and vessels. (I–L) The correlation analysis of c-JUN with Monocytes, basophils, and vessels.

Immune system disorders are important in AAN. To further compare the differences in proportions among immune cells between AAN and control tissues, we examined the GSE136276 dataset using R package mcpcounter, as presented in Fig. 6A. Monocytes, basophils, and vessels were more abundant in kidney tissues of AAN tissues than in controls, while eosinophils and endothelial cells were less expressed in the AAN tissues than in the control group. Immune activation and inhibition require a synergistic interaction among multiple cells types. To this end, we sought to understand the correlations among these immune cells. The results revealed weak correlations in immune cells in normal tissues relative to those in AAN tissues. For example, in the AAN group, there is a strong correlation between monocytes and CD8 T cells, as well as between B-derived cells. Similarly, there is a strong correlation between vessels and mast cells. (Fig. 6B, C). Furthermore, we performed the relationship between hub TFs and immune cells in AAN, evaluated by Pearson correlation (Fig. 6D–L). ATF3 and c-JUN expressions were positively correlated with monocytes, basophils, and vessels, while they negatively correlated with eosinophils and endothelial cells.

### 3.5 Hub TFs are upregulated in the kidneys of AAN mice

**Fig. 7:**
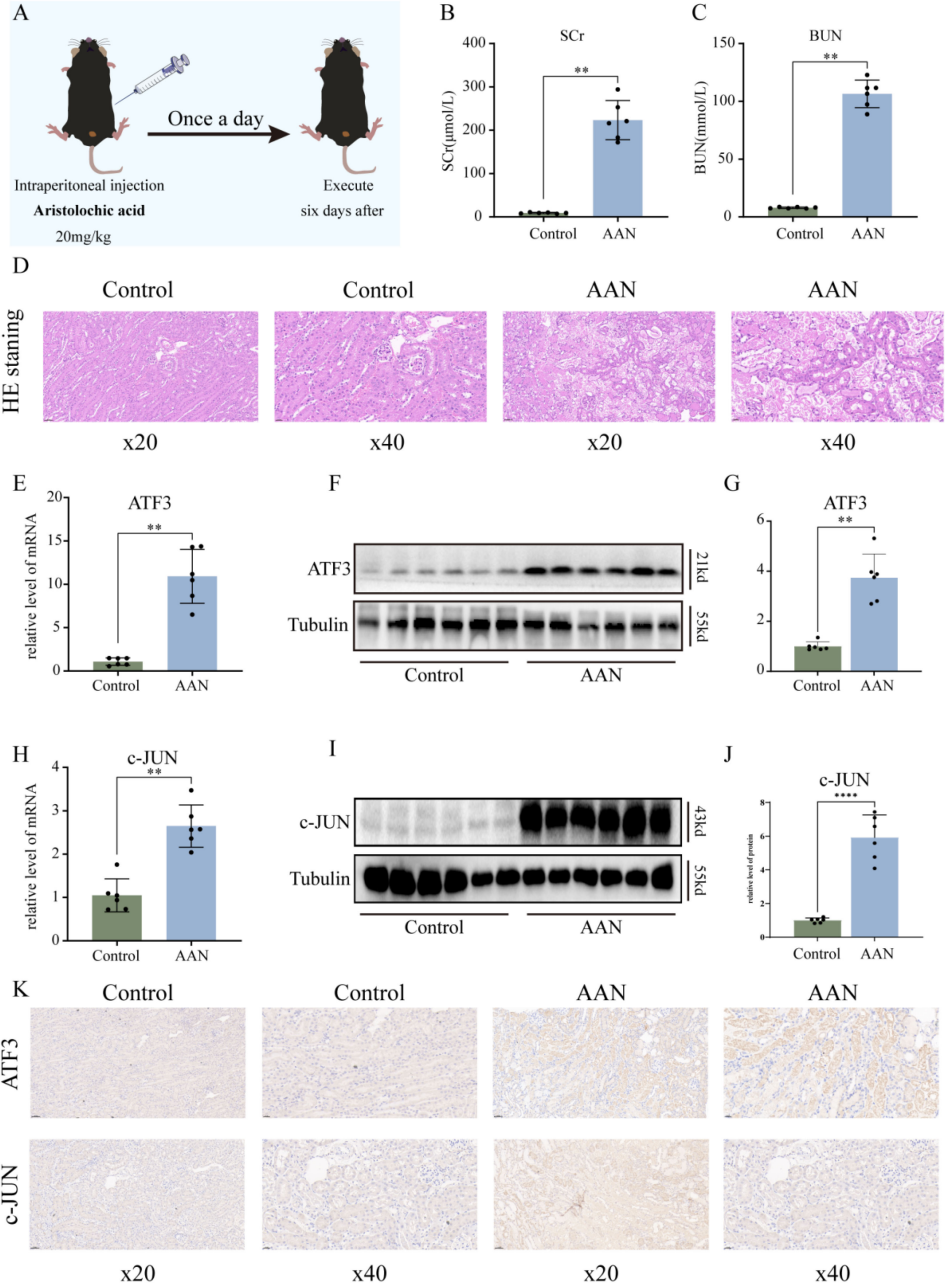
Validation of hub TFs in vivo. (A) Flow chart of AAN model construction. (B) Compared to controls, Scr levels in AAN mice, (C) Compared to controls, BUN levels in AAN mice. (D) Representative picture of HE staining. (E) The mRNA expression level of ATF3 in renal tissues of AAN mice. (F) Representative pictures of the ATF3 protein levels in renal tissue. (G) Densitometric analysis of ATF3 levels on western blots. (H) The mRNA expression level of c-JUN in renal tissues of AAN mice. (I) Representative pictures of c-JUN protein levels in renal tissues. (J) Densitometric analysis of c-JUN levels on western blots. (K) Immunohistochemical analysis of ATF3 and c-JUN levels in renal tissues. Scale bar: BUN, blood urea nitrogen, Scr, serum creatinine. Each result was repeated at least three times. Compared with the control group, *p < 0.05; **p < 0.01.

To validate the expression of hub TFs in vivo, we established an AAN mice model using our lab’s established technique (Fig. 7A). Model success was confirmed by measuring blood urea nitrogen (BUN) and Serum creatinine (Scr) levels (Fig. 7B–C), along with HE staining illustrating renal damage due to AA (Fig. 7D). Successful AKI model construction was further confirmed. By extracting kidney tissues mRNA from AAN mice, we verified increased expression of hub TFs in vivo. mRNA results revealed elevated ATF3 and c-JUN expression in AAN groups compared to controls (Fig. 7E, H). Similarly, kidney protein extraction showed significant increases in ATF3 and c-JUN consistent with mRNA levels (Fig. 7F–G, I–J). Immunostaining also displayed upregulation of ATF3 and c-JUN in the AAN mouse model (Fig. 7K). These findings collectively suggested elevated ATF3 and c-JUN expression in AAN mice, potentially playing a pivotal role in AAN development.

### 3.6 Hub TFs are upregulated by AAs in HK-2 cells

**Fig. 8:**
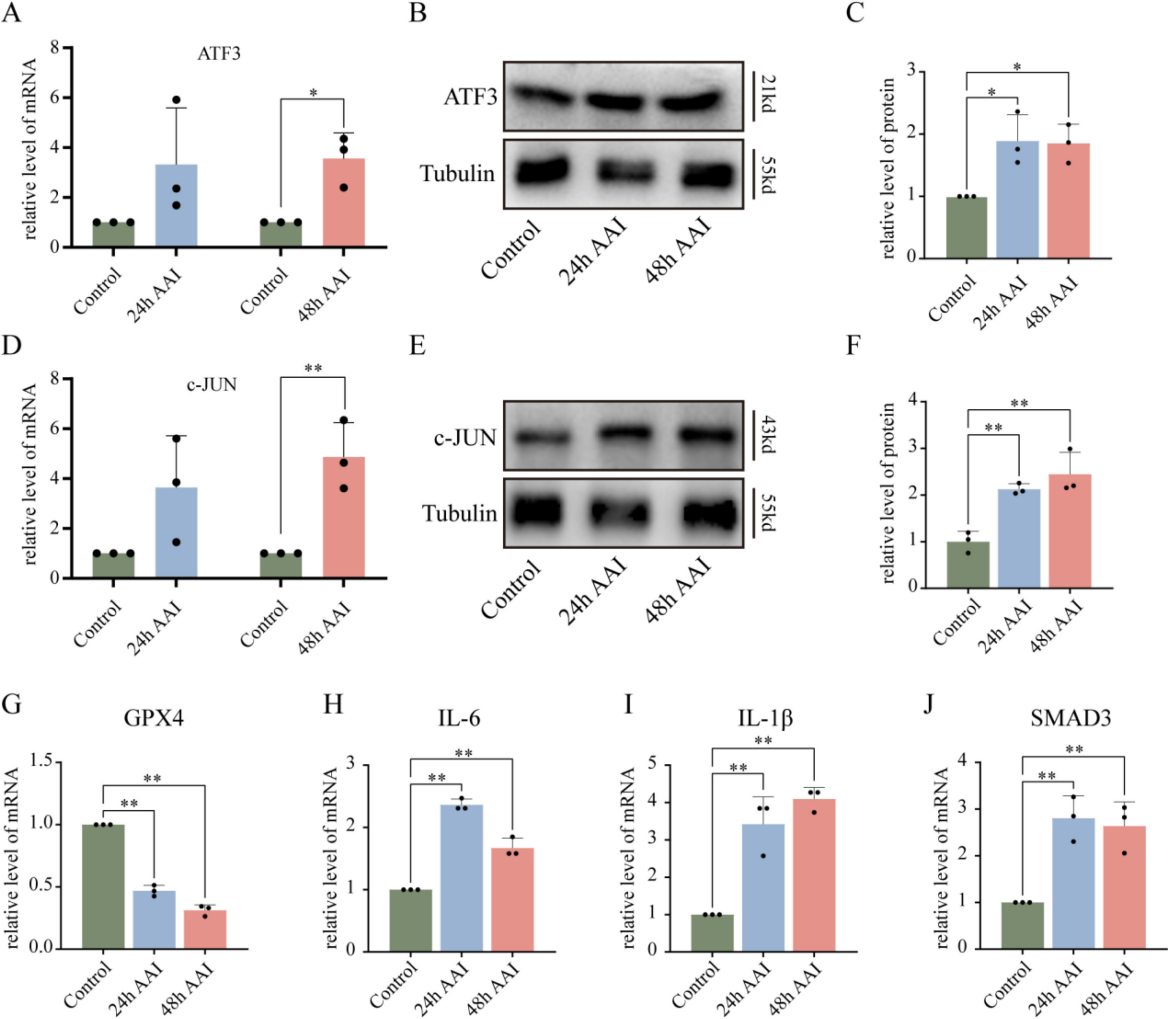
Validation of hub TFs in vitro. (A) mRNA expression of ATF3 in AA-treated cells vs. controls. (B) Representative images of ATF3 protein levels in vitro AAN. (C) Densitometric analysis of western blot ATF3 levels. (D) mRNA expression of ATF3 and c-JUN under AA treatment compared to controls. (E) Representative images of c-JUN protein levels in vitro AAN. (F) Densitometric analysis of western blot c-JUN levels. (G–J) mRNA expression of GPX4, IL-6, IL-1β, and SMAD3 under AA treatment vs. controls. AA, aristolochic acid. Each result was replicated ⩾3 times. *p < 0.05; **p < 0.01 compared to control group.

To examine the expression of two hub TFs in HK-2 cells, we determined the protein and mRNA levels of ATF3 and c-JUN. As shown in Fig. 8A–F, AA significantly increased their expression levels. Then we further validated their downstream target proteins and genes, such as GPX4, IL-6, IL-1β, and SMAD3. The real-time PCR results showed that the expression of GPX4, IL-6, IL-1β, and SMAD3 in AAN groups were significantly up-regulated compared with the control group (Fig. 8G–J). These findings indicated that the hub TFs may play a significant role in the AAN model.

### 3.7 Knockdown of ATF3 attenuates oxidative stress and inflammation

**Fig. 9:**
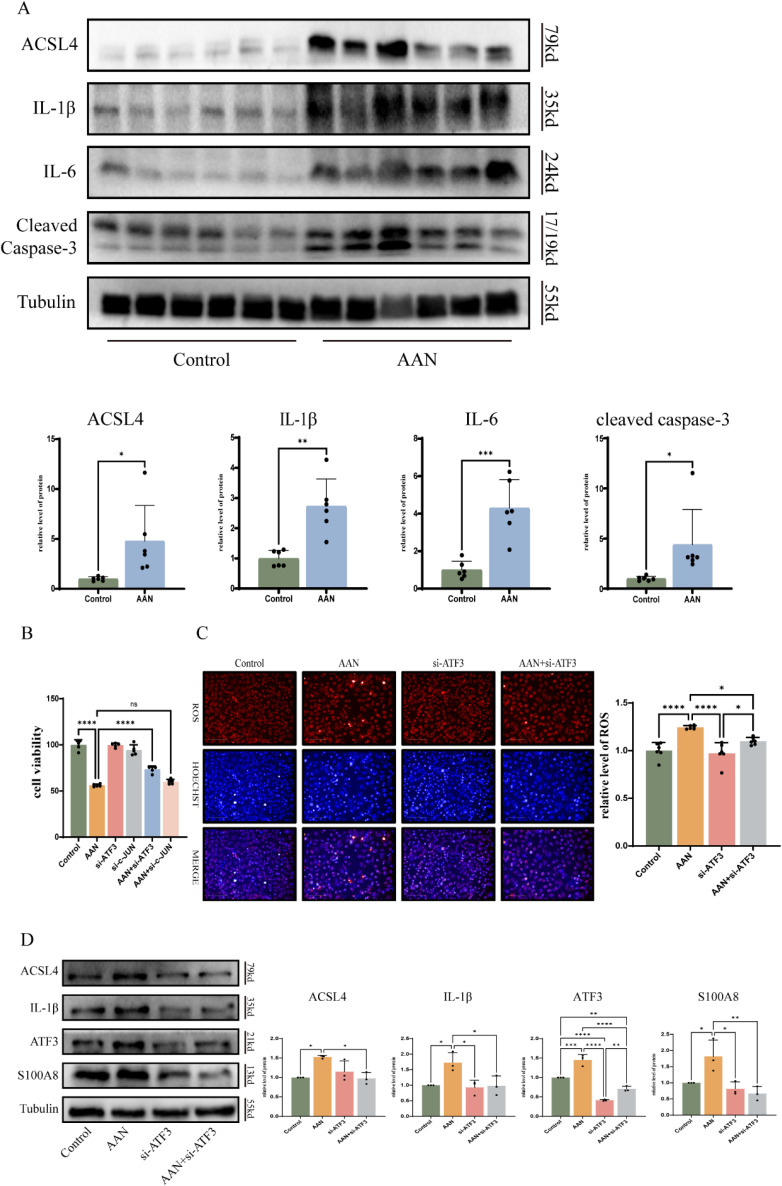
Knockdown of ATF3 attenuates oxidative stress and inflammation. (A) Proteins expression of ACSL4, IL-1β, IL-6, and cleaved Caspase-3 in in vitro AAN vs. controls. (B) The cell viability images of si-ATF3 and si-c-JUN in AA-treated cells. (C) Detection of reactive oxygen species under si-ATF3 treatment. (D) Protein expression of ACSL4, IL-1β and S100A8 after si-ATF3 treatment under AA treatment compared to controls. Each result was replicated ⩾3 times. *p < 0.05; **p < 0.01 compared to control group.

Studies have shown that apoptosis, oxidative stress, and inflammation are involved in the development and progression of AAN [9], and our mouse model results confirm this view (Fig. 9A). To confirm the effect of hub TFs in AAN, we used the si-ATF3 and si-JUN to investigate whether the knockdown of ATF3 and JUN could protect against AAN. The cell viability assay showed that the knockdown of ATF3 significantly protected against AAs-induced cell death. However, the knockdown of JUN did not alleviate cell death (Fig. 9B). We further investigated how ATF3 induced cell injury. As shown in Fig. 9C, ATF3 siRNA markedly reduced the intracellular ROS. Next, we found that knockdown of ATF3 significantly decreased the expression of ACSL4, IL-1β and S100A8, and cleaved-caspase3 induced by AA in HK-2 cells (Fig. 9D). These results indicate that ATF3 might mediated the oxidative stress and inflammation in AAN.

## 4. Discussion

AAN is a rapidly progressive interstitial nephropathy caused by AA. It mainly derived from herbal aristolochia and Asarum plants, and was listed as a human carcinogen class I in 2002 [17]. Most patients with AAN rapidly deteriorate to end-stage renal disease (ESRD) [18]. Intake of AAI was highly associated with chronic kidney disease (CKD) incidence [19]. However, products containing AA are still available, species of aristolochia are still used in medicinal preparations in many parts of the world [8]. Previous studies have showed that AA-mediated nephrotoxicity is associated with oxidative stress, apoptosis, inflammatory process in vitro and in vivo and fibrosis [20]. However, the molecular pathogenesis of AAN remains unclear. In our study, we predicted the hub TFs through bioinformatics analysis and found that ATF3 play a significant role in AAN by regulating oxidative stress, inflammation and apoptosis.

With the rapid development of high-throughput detection techniques and various databases, bioinformatics analysis is increasingly widely used to find hub genes in the human disease. Lin et al. using the fluorogenic derivatization-liquid chromatography-tandem mass spectrometry method found that TSP1 might be a novel biomarker in AAN [21]. Li et al. integrated transcriptomic, proteomic and metabolomic data and revealed that multiple pathways, particularly amino acid metabolism participated the development of AAN [22]. To identify the hub TFs in AAN, we identified co-DEGs in both HK-2 cells and mice with AAN from the GEO databases using bioinformatics analysis. PPI were used to predict potential interactive networks and identify the hub genes. Then, we intersected these hub genes with TF datasets and obtained ATF3 and c-JUN as hub TFs. Additionally, we detected hub TFs might interact with immune cells in mice model. Finally, the expression of two hub TFs were further verified in vitro and in vivo. We found that ATF3-deficient HK-2 cells protected against AA-mediated cells injury, but knockdown of c-JUN did not attenuate the cell injury. c-JUN, the most extensively studied protein of the activator protein-1 (AP-1) complex, is involved in numerous cell activities, such as proliferation, apoptosis, survival, tumorigenesis and tissue morphogenesis [24]. In human renal disease, it has been reported that c-JUN is activated in glomerular and tubular cells [23]. Yang et al. demonstrated that c-JUN amino terminal kinase (JNK) signaling pathway induced a pro-fibrotic response in AAN [24]. In our study, c-JUN is upregulated in AAN, but silencing c-JUN did not restore the cell damage. These findings suggest that c-JUN did not mediate the crucial effect of cell damage induced by AA.

TFs represent the convergence point of multiple signaling pathways, and they play a crucial role in the pathogenesis of human diseases, thus holding great therapeutic potential [25]. Several studies have demonstrated that TFs participate in the development of AAN. Smad 3 knockout mice attenuated AA-induced progressive renal dysfunction and tubulointerstitial fibrosis [26]. The transcription factor Twist1 in the distal nephron propagates chronic inflammation and fibrogenesis during AA-induced nephropathy [27]. However, no research has proposed the effect of ATF3 in AAN. Activating transcription factors, ATFs, are a family of TFs that activate gene expression and transcription by recognizing and combining the cAMP response element binding proteins (CREB) [28]. ATF3 plays a role in the cellular adaptive-response network. Multiple extracellular signals, such as endoplasmic reticulum (ER) stress, cytokines, chemokines, and lipopolysaccharide (LPS), are connected to ATF3 induction [29]. Study has shown that formononetin (FN) alleviates CKD by suppressing the Smad3/ATF3/SLC7A11 signaling [30]. Also, Quercetin can reduce ATF3 expression and further influence the downstream signaling pathway of ferroptosis, thereby ameliorate AKI induced by ischemia-reperfusion (I/R) or folic acid (FA) [31]. Our results also demonstrate that ATF3 was highly expressed in AAN, and knockdown of ATF3 protected against AA-induced cell damage.

It is well recognized that renal inflammation and oxidative stress play a significant role in AAN [20, 32]. The antioxidant lycopene protects against AAN by alleviating renal oxidative stress and attenuating renal inflammatory response and apoptosis [33]. Through pathway enrichment analysis, we found that “Toll-like receptor signaling pathway” and “Response to reactive oxygen species” participated in the development of AAN. And the proteins expression related to inflammation and apoptosis were upregulated in kidney treated with AA. Interestingly, knockdown of ATF3 prevented the ROS production in AAN cell model and attenuated AA-enhanced expression of IL-1β, ACSL4 and S100A8. These results suggested ATF3 contributes to the inflammation and oxidative stress in AAN.

However, there are some limitations of this study. We only conducted knockdown of ATF3 at cellular level to confirm its importance. Additionally, the specific mechanism of ATF3 regulating inflammation and oxidative stress remains unclear. In summary, our study demonstrates that ATF3 plays a significant role in AAN. Through bioinformatics analysis and validation in cell and animal experiments, we found that ATF3 participated in AA induced inflammation and oxidative stress. Therefore, ATF3 may be a potential therapeutic target for protecting against kidney damage induced by AA.

## Declarations

### Availability of data and materials

The datasets used in this study can be obtained by contacting the corresponding author upon reasonable request.
